# Has gene duplication impacted the evolution of Eutherian longevity?

**DOI:** 10.1111/acel.12503

**Published:** 2016-07-04

**Authors:** Aoife Doherty, João Pedro de Magalhães

**Affiliations:** ^1^ Integrative Genomics of Ageing GroupInstitute of Ageing and Chronic DiseaseUniversity of LiverpoolLiverpoolUK

**Keywords:** aging, bowhead whale, lifespan, mammals, naked mole rat

## Abstract

One of the greatest unresolved questions in aging biology is determining the genetic basis of interspecies longevity variation. Gene duplication is often the key to understanding the origin and evolution of important Eutherian phenotypes. We systematically identified longevity‐associated genes in model organisms that duplicated throughout Eutherian evolution. Longevity‐associated gene families have a marginally significantly higher rate of duplication compared to non‐longevity‐associated gene families. Anti‐longevity‐associated gene families have significantly increased rate of duplication compared to pro‐longevity gene families and are enriched in neurodegenerative disease categories. Conversely, duplicated pro‐longevity‐associated gene families are enriched in cell cycle genes. There is a cluster of longevity‐associated gene families that expanded solely in long‐lived species that is significantly enriched in pathways relating to 3‐UTR‐mediated translational regulation, metabolism of proteins and gene expression, pathways that have the potential to affect longevity. The identification of a gene cluster that duplicated solely in long‐lived species involved in such fundamental processes provides a promising avenue for further exploration of Eutherian longevity evolution.

Within Eutherians, bowhead whales (BWH) live more than 200 years, while short‐lived rodents generally live up to 4 years (de Magalhães & Costa, 2009). Gene duplication is a major player in evolution. Many longevity‐associated pathways evolved via gene duplication (Ritter *et al*., 2013), and duplication can increase lifespan (Tissenbaum & Guarente, 2001) and impact the pathogenesis of various aging‐related diseases. With the availability of genomes from long‐ and short‐lived species and a set of hundreds of genes known to influence lifespan in model organisms (LAGs = longevity‐associated genes; see Appendix S1), we thoroughly investigated the role that gene duplication played in the evolution of Eutherian longevity from a systematic perspective.

Gene duplication and loss patterns were inferred across the Eutherian phylogeny (see Appendix S1, Fig. 1). The average duplication and loss rate (DLR; measured in duplications and losses/gene/million years, DL/G/MY) was marginally significantly higher for longevity‐associated gene families (LAGFs) (0.0016 DL/G/MY) compared to non‐LAGFs (0.0012 DL/G/MY) (P = 0.0601; Mann–Whitney two‐sided test, Table S4); 230 pro‐LAGFs and 270 anti‐LAGFs were subsequently extracted based on the genes’ effects on longevity in model organisms (see Appendix S1). The average DLR in pro‐LAGFs (0.0010 DL/G/MY) is significantly lower than the DLR of the anti‐LAGFs (0.0021 DL/G/MY) (P = 0.01208; Mann–Whitney two‐sided test). The 23 pro‐ and 30 anti‐LAGFs that displayed a significant (FDR < 0.05) deviation from a random pattern of duplication and loss were extracted. One KEGG pathway was enriched in the duplicated pro‐LAGs (cell cycle; FDR = 9.7E‐03, Table S16). In the duplicated anti‐LAGs, the ribosome KEGG pathway was significantly enriched (FDR = 2.7E‐10) and two aging‐related diseases were marginally significantly enriched, Alzheimer's disease (FDR = 0.06) and Parkinson's disease (FDR = 0.09) (Table S16). Of the 52, 27 (52%) human duplicated anti‐LAGs are ohnologs (the result of a whole‐genome duplication), while 23 (61%) of the 38 pro‐LAGs are ohnologs, although a two‐sample test for equality of proportions cannot reject the null hypothesis that the two proportions are equal (P = 0.5508). Thus, there is no significant difference between the proportion of pro‐ and anti‐LAGs that duplicated via whole‐genome duplication (WGD).

**Figure 1:**
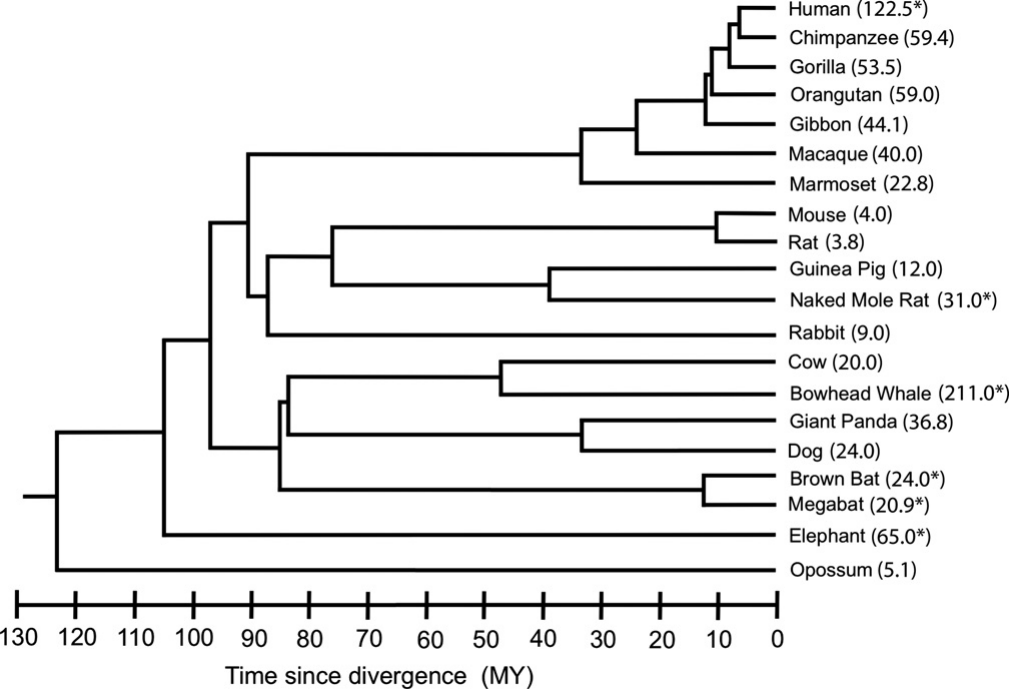
Phylogeny of species included in analysis. Longevity is indicated in brackets; long‐lived species are marked with an asterisk.

Five LAGFs experienced a significant expansion of genes in long‐lived species, while these gene numbers have remained constant in short‐lived species over time (i.e., the ‘strictly duplicated gene families’ case, see Appendix S1, Table 1, Table S5). The duplicated LAGs in these families are enriched in the Reactome biological pathways 3‐UTR‐mediated translational regulation, metabolism of proteins and gene expression, and in the GO categories related to RNA binding (Tables S6, S7, S8, & S15).

**Table 1:** Gene families with a significant expansion in only long‐lived species

Ensembl Gene Family	Gene Family Description	Species	# Genes in Species	# Genes in Species Ancestor
ENSFM00730001521743	GATA transcription factor	Brown Bat	10	4
ENSFM00500000269882	Phosphatase regulatory subunit	Human	2	1
ENSFM00250000002233	Eukaryotic initiation factor 2 (EIF2)	Bowhead Whale	7	3
		Naked Mole Rat (NMR)	4	2
ENSFM00570000851024	Polyadenylate binding PABP	Bowhead Whale	14	7
		Human	8	6
ENSFM00250000001786	60S ribosomal L10	Bowhead Whale	29	6

It is difficult to pinpoint the exact mechanisms through which these duplicated LAGs may exert an effect on longevity evolution, although it is encouraging that these pathways have previously been implicated in longevity and aging. miRNAs are involved in 3′ UTR translational regulation through binding mRNA to a 3′ UTR region of a gene to repress translation or promote mRNA degradation. The repression of translation in aging and longevity‐related networks has previously been implicated in the aging process (Chen *et al*., 2010; Mehi *et al*., 2014). It is possible that the duplication of genes involved in these processes could impact the evolution of longevity or of other life history traits. In agreement with Tacutu *et al*. (2010), a network comprising LAGs and their interactors has a higher betweenness and closeness than the full interactome (Table S9; see Appendix S1). Duplicated LAGs have a significantly higher average betweenness and closeness than all of the networks that it was compared to (i.e., the aging network, the aging network randomizations, the full interactome, and the full interactome randomizations) (Table S9; see Appendix S1). In summary, LAGs that solely duplicated in long‐lived species, while remaining conserved in copy number in short‐lived species, belong to a functional and interactomic cluster comprising central genes related to fundamental RNA‐based pathways.

All of the human genes in the strictly duplicated gene families (see Appendix S1) are classed as anti‐longevity (Table S10; relaxed case Table S11). Perhaps this is unsurprising, given the small sample size (n = 5) and that almost twice as many LAGs are assigned as anti‐longevity compared to pro‐longevity. There are a number of reasons why a LAG presumed to be anti‐longevity in a nonvertebrate model would duplicate and be retained in a long‐lived Eutherian species (Kuningas *et al*., 2008; de Magalhães, 2014). Often a mammalian genome contains several homologs of an invertebrate gene, hindering the assessment of the longevity effect of a specific gene in complex mammalian genomes. Alternatively, perhaps long‐lived species increased gene dosage of pro‐LAGs through other mechanisms, such as increasing gene expression. Finally, at least some of the genes assigned as anti‐longevity may actually exhibit pro‐longevity effects (e.g., Steffen *et al*., 2008; Hahn *et al*., 2010). As the LAGs that duplicated solely in long‐lived species were classified as anti‐longevity and comprise a functional and interactomic cluster, perhaps these genes were ohnologs (i.e., the result of a WGD event). In this case, all of the genes would duplicate simultaneously and so avoid any dosage imbalance/anti‐longevity effect that would possibly occur by duplicating these genes via small‐scale duplication. Almost all of the 17 duplicated LAGs with ohnolog information available are in fact the product of a WGD event (Tables S12, S13).

Recently, MacRae *et al*. (2015) reported in this journal that genome maintenance gene family numbers are highly conserved between human, mouse, and naked mole rat (NMR), with only two gene families having an increased copy number in NMR compared to the other species studied. In our study, these two genome maintenance gene families were not designated as aging related (see Appendix S1). In addition, although we also observed that the NMR had a higher copy number of genes in these two gene families compared to the other species that they studied (chimpanzee, mouse, rat, human, and guinea pig), these two gene families did not show a statistically significantly higher rate of duplication and loss overall compared to a random model of evolution (Table S17). However, there are also slight differences between the two analyses that could affect a direct comparison between the two studies.

We can only speculate whether the inferred duplication patterns in this study could have impacted Eutherian longevity. In reality, longevity evolution is probably the result of many genes exhibiting small effect sizes, meaning that pinpointing the exact source of longevity determination will be extremely difficult to detect, even in large studies. Because we know that the environment and condition of an individual play a massive role on the proteins that are being expressed, perhaps the true meaning of the reason for duplicating these genes in long‐lived species may not be fully understood from solely this perspective. However, subtle differences at the genomic level can exert a large phenotypic effect. The acquisition of vertebrate color vision is the result of a single visual pigment gene duplication (Yokoyama, 2002). It is possible that duplicating a small cluster of interrelated genes solely in long‐lived species that have core functions involved in RNA and protein metabolism could have directly or indirectly impacted their longevity through their functions in gene and protein networks. Finally, this research focuses on gene family expansion and their effect on the evolution of Eutherian evolution. Another interesting avenue for future research would be the role of gene loss during the evolution of long‐lived species. As high‐quality genomes of more long‐lived species become available, these findings can be validated, further elucidating the role of gene duplication in the evolution of Eutherian longevity.

## Sources of funding

A.D. was supported by a Wellcome Trust grant (104978/Z/14/Z) to J.P.M. and a European Molecular Biology Organization (EMBO) fellowship.

## Author contributions

A.D and J.P.M planned the project and edited the manuscript. A.D performed the analysis and wrote the manuscript.

## Conflict of interest

None declared.

## Supporting information


**Table S1** Longevity records (obtained from AnAge) and numbers of genes per species used in analysis.
**Table S2** Number of gene families used in each data set for CAFÉ analysis.
**Table S3** Error in each of the data sets, according to the CAFÉ algorithm.
**Table S4** Comparison of rates of gene duplication between longevity associated and non‐longevity associated genes.
**Table S5** Gene families with a significant expansion or contraction (relaxed case).
**Table S6** Functional enrichment of genes that duplicated only in longlived species.
**Table S7** Functional enrichment of genes that duplicated only in long lived species while allowing for other gene family movement to occur, compared to ageing related genes.
**Table S8** Functional enrichment of genes that duplicated only in longlived species while allowing for other gene family movement to occur, compared to full genome.
**Table S9** Comparison of network centralities between duplicated LAGs and other networks.
**Table S10** Strict duplicated genes and effect on longevity.
**Table S11** Relaxed duplicated genes and effect on longevity.
**Table S12** List of genes and their ohnolog status for the strict duplication inference.
**Table S13** List of genes and their ohnolog status for the relaxed duplication inference.
**Table S14** Sum of pairs score for each gene family alignment.
**Table S15** Functional enrichment of genes that duplicated only in longlived species (pathway analysis).
**Table S16** Functional enrichment of pro and anti‐ longevity duplicated genes.
**Table S17** CAFÉ Output P Values for the two gene families of interest from the Mac Rae *et al*. research.
**Table S18** Sets of longevity‐ and non‐longevity‐associated gene families in each data set.
**Appendix S1** Methods.
