# Supplementary material for: Has gene duplication impacted the evolution of Eutherian longevity?
Source: Aging Cell. 2016 Jul 4;15(5):978–80. doi: 10.1111/acel.12503 (PMC5013011; doi:10.1111/acel.12503)
Supplement: Supplementary file 1 — Table S1 Longevity records (obtained from AnAge) and numbers of genes per species used in analysis. Table S2 Number of gene families used in each data set for CAFÉ analysis. Table S3 Error in each of the data sets, according to the CAFÉ algorithm. Table S4 Comparison of rates of gene duplication between longevity associated and non‐longevity associated genes. Table S5 Gene families with a significant expansion or contraction (relaxed case). Table S6 Functional enrichment of genes that duplicated only in longlived species. Table S7 Functional enrichment of genes that duplicated only in long lived species while allowing for other gene family movement to occur, compared to ageing related genes. Table S8 Functional enrichment of genes that duplicated only in longlived species while allowing for other gene family movement to occur, compared to full genome. Table S9 Comparison of network centralities between duplicated LAGs and other networks. Table S10 Strict duplicated genes and effect on longevity. Table S11 Relaxed duplicated genes and effect on longevity. Table S12 List of genes and their ohnolog status for the strict duplication inference. Table S13 List of genes and their ohnolog status for the relaxed duplication inference. Table S14 Sum of pairs score for each gene family alignment. Table S15 Functional enrichment of genes that duplicated only in longlived species (pathway analysis). Table S16 Functional enrichment of pro and anti‐ longevity duplicated genes. Table S17 CAFÉ Output P Values for the two gene families of interest from the Mac Rae et al. research. Table S18 Sets of longevity‐ and non‐longevity‐associated gene families in each data set. Appendix S1 Methods. [file ACEL-15-978-s001.pdf]

**Title: Has gene duplication impacted the evolution of Eutherian longevity?**
Authors: Aoife Doherty & João Pedro de Magalhães

## Supporting Information

## METHODS

### Data set assembly

A data set comprising twenty mammalian species was assembled. Longevity records for each species were obtained from the AnAge database Build 13 (de Magalhães and Costa, 2009) (Figure 1; Table S1). The majority of the divergence estimates were derived from Benton et al. (2009), in addition to Cronin et al. (2013) and Seim et al. (2013) to resolve some of the more controversial nodes in the mammalian phylogeny. The long lived species were: human, both bat species, elephant, bowhead whale and naked mole rat.

The longest canonical transcripts and protein family assignments for all of the species except for bowhead whale (BWH) and naked mole rat (NMR) were obtained from Ensembl v 78 (Flicek et al. 2014). The longest canonical transcripts of protein coding genes for the naked mole rat and bowhead whale were obtained from the www.naked-mole-rat.org database (v 1.1) and www.bowhead-whale.org (v 1.0) respectively. In total there were 405,958 genes in the database (Table S1). NMR and BWH genes were assigned to protein families using BLAST with a $10^{-8}$ e-value cut off (Altschul et al. 1990). According to the software that calculates the duplication and loss patterns of gene families (discussed below), ~5% of gene families had an incorrect number of genes assigned to them (Table S3). This is a relatively low percentage of gene families to be incorrectly assembled in general, and much lower than the percentages that have been reported in recent studies in high quality journals (for example, Neafsey et al. 2015 recently reported that 14% of the gene families in their study were incorrectly assembled in an article in *Science*). 299 putative human aging- and/or longevity-associated genes (http://genomics.senescence.info/genes/human.html) and 1,826 human homologs to model organism aging-related genes (http://genomics.senescence.info/genes/models.html) are collectively referred to as putative longevity-associated genes (LAGs) and were obtained from GenAge Build 17 (Tacutu et al. 2013). For more information on the assignment of longevity-associated

genes, the reader is referred to Tacutu et al. (2013). Model organism genes were converted to their respective human homologs and, removing redundancy, 1,094 LAGs remained. These LAGs, combined from human and human homologs to model organism aging-related genes are referred to as the combined set LAGs. The Ensembl v78 gene family for each human protein coding gene in the genome was obtained. If a human gene was in the combined set LAGs, the gene family of this gene was assigned as a LAG family. Else, if the gene family did not contain a combined set LAG, it was assigned as a non-longevity associated gene family (non-LAG family). In total, 1,094 combined set LAGs were assigned to 739 LAG families and 16,123 non-LAGs were assigned to 10,943 non-LAG families. The list of 1,094 combined set LAGs, and the number of genes assigned to each LAG and non-LAG family are in Table S18.

**Identification of gene duplication patterns**

Once a data set had been assembled, gene families in which duplication and loss had occurred were identified. CAFÉ v 3.0 (Han et al. 2013) uses maximum likelihood to identify the rate of duplication and loss (DLR) occurring in the data set and to calculate the ancestral sizes of the gene families. Against a null hypothesis that a gene family is evolving randomly, the likelihood of the gene family size is compared to a distribution of likelihoods generated by randomly evolving n gene families (in this study, n=10,000) over the tree with the same model using a Monte Carlo approach. For families that appear to be evolving non-randomly, the specific lineage leading to the model violation is identified by comparing the likelihood of each branch to the randomly generated likelihood distribution. A P-Value for transitions between parent and child nodes for all branches of the tree is obtained. A Benjamini-Hochberg (i.e. FDR) cut off $< 0.05$ was considered to be statistically significant for each of the gene families, and $P < 0.01$ was used to identify the branch in the family that was not randomly evolving.

Five different mammalian data sets were used in this analysis in order to validate the robustness of any results obtained. First, since the CAFÉ algorithm requires gene families that include the root of the phylogenetic tree in order to accurately calculate duplication and loss patterns using the birth death process, changing the root of the tree changes the number of gene families that are included in the analysis. The root was changed twice: first by removing the opossum (i.e. the data set named "no opossum"),

and second by removing the elephant (i.e. “no elephant”). Second, two separate groups of species were randomly deleted from the data set to examine how this would affect the rates of duplication and loss observed in both LAGs and non-LAGs. In one case, orang utan, guinea pig and dog were removed (i.e. “removed PPY-CPO-CAF”), and in the second case, macaque, rat and cow were removed (i.e. removed “MMU-RNO-BTA”) (Table S2; Table S18 for all genes assigned to all LAG and non-LAG fams.).

**Assignment of duplicated genes as pro- and anti- longevity genes**

For the genes with relevant information available in GenAge, each LAG was assigned as pro- or anti- longevity, depending on the effect that was recorded in GenAge (Table S18; Tacutu et al. (2013)). Briefly, pro-longevity genes are defined as genes whose over-expression extends lifespan or whose decreased activity (e.g. because of knockout or RNA interference) decreases it. Anti-longevity genes are those for which the aforementioned interventions have the opposite effects. It is important to note that an anti-longevity gene is not equivalent to a non-longevity associated gene. A non-longevity associated gene is not (yet) known to impact an organism’s longevity; whereas an anti-longevity gene is one whose decreased expression extends lifespan and/or whose overexpression decreases it. In total, there were 230 genes assigned as pro-longevity and 270 genes assigned as anti-longevity. The CAFÉ analysis was repeated as in the previous section for the sub-set of pro- and anti- longevity associated families. The families with statistically significant patterns of gene duplication (FDR < 0.05) were extracted for further analysis. Functional enrichment analysis was conducted using DAVID (Huang et al. 2007), searching for enrichment in KEGG pathways between the gene set of interest and the full human genome.

**Identification of gene duplication mechanism for each gene**

Whole genome duplication played a vital role in the evolution of vertebrate complexity, particularly in evolving pathways with delicately balanced components that require constant maintenance of dosage balance among interacting genes. To understand the duplication mechanism for each paralog, each duplicated LAG was described as either an ohnolog (the result of a whole genome duplication) or not, according to the methods of Singh et al. (2015) (Table S12, S13).

**Extraction of gene families of interest for further analysis**

To understand how gene duplication could have aided the evolution of mammalian longevity, families with statistically significant higher rates of duplication in long-lived species than expected were extracted for further analysis. The results reported here refer to the data set that excludes the genetic data from the opossum. The reason for this was to solely focus on the duplication patterns observed in the well-sampled placental mammal group, since the opossum is the only marsupial in the data set. However, the analysis was also conducted using the information from the opossum, with almost identical results. The manuscript focuses on the more interesting "strict" case, where the only movement in gene family size has been a significant expansion of genes in only long lived species, while in all short lived species, gene numbers have remained constant over millions of years. These are referred to in the main text as "strictly duplicated gene families" (i.e. Table 1). In addition, we also considered a "relaxed" approach, where there was significant expansion or contraction in long lived species, while, unlike the "strict" case, there was also allowed to be expansion and contraction in the non-long lived species. Where appropriate, relevant details about the "relaxed" approach, which allowed for gene family movement in short lived species, is also described.

The quality of the inferred duplications for the case of interest, the strict case, were checked using multiple approaches. First, CAFÉ software suggested that ~5% of all of the gene families had incorrect gene numbers assigned to it. This is quite low in general, and lower than the error assigned to gene families for some recent high quality publications (e.g. Neafsey et al. 2015). Second, all of the duplicated genes that are deposited in the Ensembl database (i.e. the human and bat paralogs) in the families of interest were checked to ensure that these duplicated genes actually produced protein products, and were not pseudogenes. Third, for each gene family, multiple alignments were constructed and a sum-of-pairs score was calculated for each alignment. The average sum-of-pairs score for the alignments is 390.76 and almost all gene families looked well aligned upon visual inspection (Table S14). However one of the gene families, 60S Ribosomal L10, had a sum-of-pairs score far below the average (151.3). This would indicate that this particular family may be poorly aligned and may have an incorrect number of genes, particularly belonging to the newly sequenced Bowhead

Whale genome, attached to it. In summary, the majority of the gene families appear to be well constructed and the inferred duplications may be considered to be of reasonably high quality. Perhaps some extra caution should be taken in the interpretation of the Bowhead Whale duplicated genes, although considering that this genome assembly is quite novel compared to almost all of the other genomes in this study, such caution should not be restricted to solely this study.

**Functional enrichment analysis**

We wanted to understand if the set of duplicated genes in long-lived species were enriched in particular functions. The 17 human genes in the duplicated LAG families (and 149 human genes in the LAG families in the relaxed case) of interest were compared to both the full human genome, and to the full set of longevity associated genes using DAVID (Huang et al. 2007), searching for enrichment in the categories: cellular component, biological process, molecular function and KEGG pathway.

**Protein protein interaction analysis**

To further investigate how the duplicated genes function, we examined where the duplicated genes are located in the protein interaction network (PIN), relative to the full network of ageing related genes, and the full human PIN. For this, the human protein protein interaction network was obtained from BioGrid version 3.3.124 (Chatr-Anyamontri et al. 2014). After filtering to retain non-redundant physical interactions between human species that were not self-interactions, there were 157,635 interactions. The 17 human genes that solely duplicated in long-lived species (i.e. the "strict" case) were directly involved in 545 interactions in this interactome. Allowing for interactions between the interactors of the 17 human genes, there were 8,652 interactions in the "strict duplicated network". Then, an ageing network of LAGs and their interactions contained 152,231 edges.

The network centralities of interest are average betweenness (i.e. the number of shortest paths from all vertices to all other vertices that pass through that node) and closeness (i.e. the inverse of the sum of the distances from a node to all other nodes). These were calculated using the NetworkX package v. 1.9.1 (http://networkx.lanl.gov/). Network centralities for the subnetwork of duplicated LAGs and their interactors was

compared to (1) the ageing network, (2) 100 randomised ageing related networks, (3) The full human interactome and (4) 100 randomised full human interactome (Table S9). The statistical significance of the measured network parameters for the set of duplicated genes compared to the other networks was evaluated with an ensemble of 100 randomised networks that were generated using the network rewiring approach as described in Doherty et al. (2012).

**Comparison to MacRae et al. (2015) research**

In the manuscript, we describe a comparison to recently published research from MacRae et al. (2015). The specific genes studied by MacRae et al. (2015) have not, as of yet, been officially identified as longevity associated based on the GenAge curation mechanism (i.e., genetic manipulations in model systems, such as mice, demonstrating a significant impact on aging and/or longevity) and therefore were not assigned as longevity associated in this analysis. However, the gene families were available in the non-LAG dataset (Table S18), and so the duplication pattern observed in these families were still calculated by the CAFÉ software.

**Supplementary Table 1.** Longevity records (obtained from AnAge) and numbers of genes per species used in analysis.

| Species | Longevity (*=Long lived) | Number of genes |
|---:|---:|---:|
| **Chimpanzee** | 59.4 | 18,759 |
| **Cow** | 20.0 | 19,994 |
| **Dog** | 24.0 | 19,856 |
| **Elephant** | 65.0* | 20,333 |
| **Gibbon** | 44.1 | 18,575 |
| **Gorilla** | 53.5 | 20,962 |
| **Guinea pig** | 12.0 | 18,673 |
| **Human** | 122.5* | 19,079 |
| **Macaque** | 40.0 | 21,905 |
| **Marmoset** | 22.8 | 20,978 |
| **Flying Fox** | 20.9* | 16,990 |
| **Brown Bat** | 24.0* | 19,728 |
| **Mouse** | 4.00 | 22,196 |
| **Opossum** | 5.10 | 21,327 |
| **Orangutan** | 59.0 | 20,424 |
| **Panda** | 36.8 | 19,343 |
| **Rabbit** | 9.00 | 19,293 |
| **Rat** | 3.80 | 22,776 |
| **Naked Mole Rat** | 31.0* | 22,112 |
| **Bowhead Whale** | 211* | 22,655 |

**Supplementary Table 2.** Number of gene families used in each data set for CAFÉ analysis.

| | LAG Families | Rest of Genome Families |
|---|---|---|
| **Initial Analysis** | 697 | 9,536 |
| **Change Root** | | |
| **No Opossum** | 724 | 10,287 |
| **No Elephant** | 736 | 10,880 |
| **Remove Species** | | |
| **No PPY-CPO-CAF** | 697 | 9,536 |
| **No MMU-RNO-BTA** | 697 | 9,536 |

**Supplementary Table 3.** Error in each of the data sets, according to the CAFÉ algorithm.

| | LAG Families (%) | Rest of Genome Families (%) |
|---|---|---|
| **Initial Analysis** | 5.2 | 4.7 |
| **Change Root** | | |
| **No Opossum** | 4.5 | 5.3 |
| **No Elephant** | 4.0 | 6.8 |
| **Remove Species** | | |
| **No PPY-CPO-CAF** | 7.0 | 7.9 |
| **No MMU-RNO-BTA** | 5.3 | 4.4 |

**Supplementary Table 4**. Comparison of rates of gene duplication between longevity associated and non-longevity associated genes.

| | LAGs (DL/G/MY) | Rest of Genome (DL/G/MY) |
|---|---|---|
| **Initial analysis** | 0.0015 | 0.0013 |
| **Change Root** | | |
| **No Opossum** | 0.0018 | 0.0012 |
| **No Elephant** | 0.0021 | 0.0013 |
| **Remove Species** | | |
| **No PPY-CPO-CAF** | 0.0012 | 0.0011 |
| **No MMU-RNO-BTA** | 0.0013 | 0.0012 |

**Supplementary Table 5.** Gene families with a significant expansion or contraction (relaxed case).

| Ensembl Gene Family | Species | # Genes in species | # Genes in species ancestor |
|---|---|---|---|
| ENSFM00250000000455 | Brown Bat | 6 | 4 |
| | Bowhead Whale | 8 | 4 |
| ENSFM00250000001425 | Bowhead Whale | 3 | 1 |
| ENSFM00250000001600 | Megabat | 8 | 5 |
| ENSFM00250000001786 | Bowhead Whale | 29 | 6 |
| ENSFM00250000001949 | Bowhead Whale | 6 | 3 |
| ENSFM00250000002233 | Bowhead Whale | 7 | 3 |
| | Naked Mole Rat | 4 | 2 |
| ENSFM00250000002321 | Brown Bat | 4 | 2 |
| | Bowhead Whale | 11 | 3 |
| ENSFM00250000003189 | Bowhead Whale | 7 | 1 |
| ENSFM00250000003294 | Brown Bat | 5 | 2 |
| ENSFM00270000056431 | Brown Bat | 15 | 12 |
| | Bowhead Whale | 39 | 19 |
| ENSFM00270000056439 | Brown Bat | 6 | 4 |
| ENSFM00350000105437 | Bowhead Whale | 9 | 3 |
| ENSFM00500000269788 | Bowhead Whale | 8 | 4 |
| ENSFM00500000269882 | Human | 2 | 1 |
| ENSFM00500000270597 | Bowhead Whale | 9 | 4 |
| ENSFM00500000271122 | Brown Bat | 13 | 2 |
| ENSFM00570000851024 | Bowhead Whale | 14 | 7 |
| | Human | 8 | 6 |
| ENSFM00670001235396 | Bowhead Whale | 8 | 2 |
| | Brown Bat | 6 | 2 |
| ENSFM00670001235422 | Bowhead Whale | 14 | 7 |
| ENSFM00670001235586 | Brown Bat | 6 | 3 |
| | Bowhead Whale | 13 | 4 |
| ENSFM00670001236147 | Bowhead Whale | 4 | 1 |
| ENSFM00730001521078 | Bowhead Whale | 43 | 16 |
| ENSFM00730001521103 | Bowhead Whale | 15 | 6 |
| ENSFM00730001521154 | Bowhead Whale | 17 | 9 |
| ENSFM00730001521252 | Human | 29 | 25 |
| | Brown Bat | 62 | 27 |
| ENSFM00730001521743 | Brown Bat | 10 | 4 |
| ENSFM00750001632319 | Megabat | 11 | 7 |
| ENSFM00750001632341 | Brown Bat | 9 | 6 |
| | Bowhead Whale | 13 | 8 |
| ENSFM00760001714593 | Megabat | 23 | 18 |
| ENSFM00760001714648 | Bowhead Whale | 8 | 2 |

**Supplementary Table 6.** Functional enrichment of genes that duplicated only in long-lived species.

| GO Definition | FDR |
|---|---|
| **Comparison between duplicated LAGs and Full Genome** | |
| Poly(A) RNA Binding | 8.8e-04 |
| Poly-purine tract binding | 5.6e-04 |
| Single stranded RNA binding | 3.6e-03 |
| RNA binding | 3.8e-03 |
| **Comparison between duplicated LAGs and Full set of LAGs** | |
| Poly-purine tract binding | 6.0e-03 |
| Poly(A) RNA binding | 6.0e-03 |

**Supplementary Table 7.** Functional enrichment of genes that duplicated only in long lived species while allowing for other gene family movement to occur, compared to ageing related genes.

| GO Definition | FDR |
|---|---|
| Ribonucleoprotein complex | 1.10E-15 |
| Regulation of RNA metabolic process | 4.40E-08 |
| Zinc ion binding | 1.40E-07 |
| Regulation of transcription, DNA-dependent | 1.70E-07 |
| Regulation of transcription | 7.20E-07 |
| Transcription | 8.70E-07 |
| DNA binding | 2.10E-06 |
| Cytosolic ribosome | 3.00E-06 |
| Ribosomal subunit | 1.10E-05 |
| Cytosolic large ribosomal subunit | 1.40E-05 |
| Ribosome | 1.40E-05 |
| Transition metal ion binding | 1.50E-05 |
| Translational elongation | 6.90E-05 |
| Large ribosomal subunit | 1.00E-04 |
| Cytosolic part | 1.20E-04 |
| RNA binding | 1.70E-04 |
| Heterogeneous nuclear ribonucleoprotein complex | 1.90E-04 |
| Structural constituent of ribosome | 5.90E-04 |
| Cytosol | 1.70E-03 |
| Spliceosome | 1.90E-03 |
| Metal ion binding | 3.50E-03 |
| Ion binding | 4.20E-03 |
| Cation binding | 4.30E-03 |
| mRNA binding | 6.00E-03 |

**Supplementary Table 8.** Functional enrichment of genes that duplicated only in long-lived species while allowing for other gene family movement to occur, compared to full genome.

| GO Description | FDR |
|---|---|
| Regulation of RNA metabolic process | 4.80E-33 |
| Regulation of transcription, DNA-dependent | 6.40E-32 |
| Regulation of transcription | 1.70E-28 |
| Transcription | 2.50E-28 |
| Zinc ion binding | 1.80E-22 |
| Transition metal ion binding | 1.80E-18 |
| Ribonucleoprotein complex | 5.50E-16 |
| Metal ion binding | 2.00E-10 |
| Cation binding | 2.70E-10 |
| Ion binding | 5.10E-10 |
| Cytosolic ribosome | 1.80E-08 |
| Ribosome | 3.10E-08 |
| Cytosolic part | 8.40E-08 |
| Cytosol | 1.30E-07 |
| Ribosomal subunit | 2.80E-07 |
| Ribosome | 8.70E-07 |
| Translational elongation | 2.00E-06 |
| Cytosolic large ribosomal subunit | 3.10E-06 |
| Heterogeneous nuclear ribonucleoprotein complex | 4.20E-06 |
| Large ribosomal subunit | 4.30E-05 |
| Spliceosome | 1.10E-04 |
| RNA binding | 2.00E-04 |
| Oocyte meiosis | 4.10E-04 |
| Structural constituent of ribosome | 4.20E-04 |
| mRNA binding | 4.40E-04 |
| Poly (A) RNA binding | 4.90E-04 |
| Translation | 5.90E-04 |
| Poly-purine tract binding | 6.60E-04 |
| Single-stranded RNA binding | 1.00E-03 |
| Intracellular non-membrane-bounded organelle | 7.30E-03 |
| Non-membrane-bounded organelle | 7.30E-03 |
| Antigen processing and presentation | 9.10E-03 |
| DNA binding | 4.70E-28 |
| Cell cycle | 6.3E0-04 |

**Supplementary Table 9.** Comparison of network centralities between duplicated LAGs and other networks**.**

| Network | Ave. Network Betweenness | Betweenness P | Ave. Network Closeness | Closeness P |
|---|---|---|---|---|
| **Original Duplicated Strict Network (8,652 edges)** | 0.0027 | - | 0.495 | - |
| **Original Duplicated Relaxed Network (45,945 edges)** | 0.0005 | | 0.457 | |
| **Full Ageing Network** | 0.0001 | - | 0.419 | - |
| **Random Ageing Networks (152,231 edges; 100 random networks)** | 0.0001 | <0.01 | 0.416 | <0.01 |
| **Full Interactome** | 0.0001 | - | 0.383 | - |
| **Random Full Interactome (157,635 edges, 100 random networks)** | 0.0001 | < 0.01 | 0.386 | <0.01 |

**Supplementary Table 10.** Strict duplicated genes and effect on longevity.

| Gene | Effect on Longevity (pro/anti) |
|---|---|
| ENSG00000102145 | anti |
| ENSG00000188386 | anti |
| ENSG00000130741 | anti |
| ENSG00000101104 | anti |
| ENSG00000165496 | anti |

**Supplementary Table 11.** Relaxed duplicated genes and effect on longevity.

| Gene | Effect on lifespan (pro/anti/fitness) |
|---|---|
| ENSG00000113558 | pro |
| ENSG00000115268 | anti |
| ENSG00000157106 | anti |
| ENSG00000108298 | anti |
| ENSG00000174444 | anti |
| ENSG00000136810 | pro |
| ENSG00000071082 | anti |
| ENSG00000163682 | anti |
| ENSG00000197728 | anti |
| ENSG00000132141 | anti |
| ENSG00000130741 | anti |
| ENSG00000137154 | anti |
| ENSG00000089009 | fitness |
| ENSG00000183791 | anti |
| ENSG00000165496 | anti |
| ENSG00000101104 | anti |
| ENSG00000101210 | pro |
| ENSG00000102145 | anti |
| ENSG00000188386 | anti |
| ENSG00000139675 | anti |
| ENSG00000133661 | anti |
| ENSG00000198947 | pro |
| ENSG00000152818 | pro |
| ENSG00000166913 | pro |
| ENSG00000138668 | pro |

**Supplementary Table 12.** List of genes and their ohnolog status for the strict duplication inference.

| **Gene** | **Ohnolog (Y/N)** |
|---|---|
| ENSG00000107485 | Y |
| ENSG00000102145 | Y |
| ENSG00000179348 | Y |
| ENSG00000221823 | Y |
| ENSG00000188386 | Y |
| ENSG00000130741 | N |
| ENSG00000180574 | N |
| ENSG00000174740 | Y |
| ENSG00000090621 | Y |
| ENSG00000151846 | Y |
| ENSG00000186288 | Y |
| ENSG00000254535 | Y |
| ENSG00000101104 | Y |
| ENSG00000184388 | Y |
| ENSG00000070756 | Y |
| ENSG00000147403 | N |
| ENSG00000165496 | N |

**Supplementary Table 13.** List of genes and their ohnolog status for the relaxed duplication inference.

| Gene | Ohnolog (Y/N) |
|---|---|
| ENSG00000196597 | N |
| ENSG00000221994 | N |
| ENSG00000176024 | N |
| ENSG00000142556 | N |
| ENSG00000161298 | N |
| ENSG00000147118 | N |
| ENSG00000198105 | N |
| ENSG00000196693 | N |
| ENSG00000196458 | N |
| ENSG00000188227 | N |
| ENSG00000198185 | N |
| ENSG00000256087 | N |
| ENSG00000145908 | N |
| ENSG00000136866 | N |
| ENSG00000141946 | N |
| ENSG00000256683 | N |
| ENSG00000075407 | N |
| ENSG00000161551 | N |
| ENSG00000189042 | N |
| ENSG00000146587 | N |
| ENSG00000189180 | N |
| ENSG00000251192 | N |
| ENSG00000198093 | N |
| ENSG00000164631 | N |
| ENSG00000197619 | N |
| ENSG00000184517 | N |
| ENSG00000081386 | N |
| ENSG00000198393 | N |
| ENSG00000113558 | N |
| ENSG00000115268 | N |
| ENSG00000157106 | N |
| ENSG00000261740 | N |
| ENSG00000108298 | N |
| ENSG00000174444 | N |
| ENSG00000136810 | N |
| ENSG00000071082 | N |
| ENSG00000163682 | N |
| ENSG00000177733 | N |

| | |
|---|---|
| ENSG00000213762 | N |
| ENSG00000121406 | N |
| ENSG00000171574 | N |
| ENSG00000269343 | N |
| ENSG00000105136 | N |
| ENSG00000083814 | N |
| ENSG00000196724 | N |
| ENSG00000197128 | N |
| ENSG00000179909 | N |
| ENSG00000131849 | N |
| ENSG00000083817 | N |
| ENSG00000152443 | N |
| ENSG00000198466 | N |
| ENSG00000186230 | N |
| ENSG00000186272 | N |
| ENSG00000171649 | N |
| ENSG00000178935 | N |
| ENSG00000188785 | N |
| ENSG00000173480 | N |
| ENSG00000180884 | N |
| ENSG00000183647 | N |
| ENSG00000204519 | N |
| ENSG00000152433 | N |
| ENSG00000152439 | N |
| ENSG00000152454 | N |
| ENSG00000131845 | N |
| ENSG00000121417 | N |
| ENSG00000159882 | N |
| ENSG00000159885 | N |
| ENSG00000263002 | N |
| ENSG00000267508 | N |
| ENSG00000167380 | N |
| ENSG00000159905 | N |
| ENSG00000267680 | N |
| ENSG00000159915 | N |
| ENSG00000149050 | N |
| ENSG00000124459 | N |
| ENSG00000204920 | N |
| ENSG00000062370 | N |
| ENSG00000256294 | N |
| ENSG00000131115 | N |
| ENSG00000196793 | N |
| ENSG00000178386 | N |
| ENSG00000159917 | N |
| ENSG00000186026 | N |
| ENSG00000278318 | N |

| | |
|---|---|
| ENSG00000197728 | N |
| ENSG00000132141 | N |
| ENSG00000146731 | N |
| ENSG00000130741 | N |
| ENSG00000180574 | N |
| ENSG00000137154 | N |
| ENSG00000089009 | N |
| ENSG00000204389 | N |
| ENSG00000204388 | N |
| ENSG00000126803 | N |
| ENSG00000204390 | N |
| ENSG00000275553 | N |
| ENSG00000274744 | N |
| ENSG00000206181 | N |
| ENSG00000011007 | N |
| ENSG00000183791 | N |
| ENSG00000144381 | N |
| ENSG00000147403 | N |
| ENSG00000165496 | N |
| ENSG00000198040 | Y |
| ENSG00000090612 | Y |
| ENSG00000174740 | Y |
| ENSG00000090621 | Y |
| ENSG00000151846 | Y |
| ENSG00000186288 | Y |
| ENSG00000254535 | Y |
| ENSG00000101104 | Y |
| ENSG00000184388 | Y |
| ENSG00000070756 | Y |
| ENSG00000101210 | Y |
| ENSG00000156508 | Y |
| ENSG00000107485 | Y |
| ENSG00000102145 | Y |
| ENSG00000179348 | Y |
| ENSG00000221823 | Y |
| ENSG00000188386 | Y |
| ENSG00000139675 | Y |
| ENSG00000122566 | Y |
| ENSG00000135486 | Y |
| ENSG00000170144 | Y |
| ENSG00000204514 | Y |
| ENSG00000083828 | Y |
| ENSG00000133661 | Y |
| ENSG00000102385 | Y |
| ENSG00000198947 | Y |
| ENSG00000152818 | Y |

| | |
|---|---|
| ENSG00000164924 | Y |
| ENSG00000134308 | Y |
| ENSG00000170027 | Y |
| ENSG00000166913 | Y |
| ENSG00000128245 | Y |
| ENSG00000175793 | Y |
| ENSG00000109971 | Y |
| ENSG00000173110 | Y |
| ENSG00000197451 | Y |
| ENSG00000138668 | Y |
| ENSG00000152795 | Y |

**Supplementary Table 14.** Sum of pairs score for each gene family alignment.

| Family | Sum of pairs score from multiple alignment (MUSCLE) |
|---|---|
| ENSFM00250000001786 | 151.3 |
| ENSFM00730001521743 | 554.4 |
| ENSFM00500000269882 | 253.4 |
| ENSFM00250000002233 | 386.8 |
| ENSFM00570000851024 | 607.9 |

**Supplementary Table 15.** Functional enrichment of genes that duplicated only in long-lived species (pathway analysis)

| Pathway Category | Pathway Term | FDR |
|---|---|---|
| Reactome Pathway | REACT_1762: 3-UTR-mediated translational regulation | 3E-03 |
| Reactome Pathway | REACT_17015: Metabolism of proteins | 6.1E-03 |
| Reactome Pathway | React_71: Gene expression | 1.1E-02 |
| KEGG Pathway | Ribosome | 3.8E-01 |

**Supplementary Table 16** Functional enrichment of pro and anti- longevity duplicated genes.

| Pathway Category | Pathway Term | FDR |
|---|---|---|
| **Pro Longevity Genes** | | |
| KEGG Pathway | Cell Cycle | 9.7E-03 |
| **Anti Longevity Genes** | | |
| KEGG Pathway | Ribosome | 2.7E-10 |
| KEGG Pathway | Alzheimer's Disease | 6.6E-02 |
| KEGG Pathway | Parkinson's Disease | 8.7E-02 |

**Supplementary Table S17** CAFÉ Output P Values for the two gene families of interest from the Mac Rae et al. research.

| Gene Family | Gene Family Description | CAFÉ P |
|---|---|---|
| CEBPG | ENSFM00500000273446 | 0.474 |
| TINF2 | ENSFM00250000008327 | 0.165 |

**Bibliography**

Altschul, S. F., Gish, W., Miller, W., Myers, E. W., & Lipman, D. J. (1990). Basic Local Alignment Search Tool. Journal of Molecular Biology, *215*, 403-410.

Benton, M. J., P. C. J. Donoghue, and R. J. Asher. (2009) Calibrating and constraining molecular clocks. The Timetree of Life, 35-86.

Chatr-Aryamontri, A., Breitkreutz, B.-J., Heinicke, S., Boucher, L., Winter, A., Stark, C., Nixon, J., Ramage, L., Kolas, N., O'Donnell, L., Reguly,, T., Breitkreutz, A., Sellam, A., Chen, D., Chang, C., Rust, J., Livstone, M., Oughtred, R., Dolinski, K. & Tyers, M. (2013). The BioGRID interaction database: 2013 update. Nucleic Acids Research, *41(Database issue)*, D816–23.

Cronin, M. A., Rincon, G., Meredith, R. W., MacNeil, M. D., Islas-Trejo, A., Canovas, A. & Medrano, J. F. (2014) Molecular phylogeny and SNP variation of polar bears (Ursus maritimus), brown bears (U. arctos), and black bears (U. americanus) derived from genome sequences. Journal of Heredity *105*, 312-323.

De Magalhães, J. P., & Costa, J. (2009). A database of vertebrate longevity records and their relation to other life-history traits. Journal of Evolutionary Biology *22*, 1770–4.

Doherty, A., Alvarez-Ponce, D. and McInerney, J. O. (2012) Increased genome sampling reveals a dynamic relationship between gene duplicability and the structure of the primate protein–protein interaction network. Molecular Biology and Evolution *29*, 3563-3573.

Flicek, P., Amode, M. R., Barrell, D., Beal, K., Billis, K., Brent, S., … Searle, S. M. J. (2014). Ensembl 2014. Nucleic Acids Research *42(Database issue)*, D749–55.

Han, M. V., Thomas, G. W. C., Lugo-Martinez, J. & Hahn, M. W. (2013) Estimating gene gain and loss rates in the presence of error in genome assembly and annotation using CAFE3. Molecular Biology and Evolution *30*, 1987-1997.

Huang, D. W., Sherman, B. T., Tan, Q., Kir, J., Liu, D., Bryant, D., Guo, Y., Stephens, R., Baseler, M., Lane, C. & Lempicki, R. A. (2007). DAVID Bioinformatics Resources: expanded annotation database and novel algorithms to better extract biology from large gene lists. Nucleic Acids Research *35(Web Server issue)*, W169–75.

MacRae, S. L., Zhang, Q., Lemetre, C., Seim, I., Calder, R. B., Hoeijmakers, J., Suh, Y., Gladyshev, V., Seluanov, A., Gorunova, V., Vijg, J. & Zhang, Z. D. (2015). Comparative analysis of genome maintenance genes in naked mole rat, mouse, and human. Aging Cell *14*, 288–91.

Neafsey, D. E., Waterhouse, R. M., Abai, M. R., Aganezov, S. S., Alekseyev, M. A., Allen, J. E., … Besansky, N. J. (2015). Mosquito genomics. Highly evolvable malaria vectors: the genomes of 16 Anopheles mosquitoes. Science (New York, N.Y.) *347,* 1258522.

Seim, I., Fang, X., Xiong, Z., Lobanov, A., Huang, Z., Ma, S. et al. (2013) Genome analysis reveals insights into physiology and longevity of the Brandt's bat Myotis brandtii. Nature communications *4* (2212).

Singh, P., Arora, J., & Isambert, H. (2015). Identification of Ohnolog Genes Originating from Whole Genome Duplication in Early Vertebrates, Based on Synteny Comparison across Multiple Genomes. PLoS Comput Biol *11*, e1004394.

Tacutu, R., Craig, T., Budovsky, A., Wuttke, D., Lehmann, G., Taranukha, D., Costa, J., Fraifeld, V. & de Magalhães, J. P. (2013). Human Ageing Genomic Resources: Integrated databases and tools for the biology and genetics of ageing. Nucleic Acids Research, *41* (Database issue), D1027–D1033.
